# Inpatient Ward Review Safety Documentation Re-audit

**DOI:** 10.1192/bjo.2022.502

**Published:** 2022-06-20

**Authors:** Oksana Zinchenko, Yasmin Meakin, Lloyd Davies, Shafalica Bhan - Kotwal

**Affiliations:** Essex Partnership University NHS Foundation Trust, Colchester, United Kingdom

## Abstract

**Aims:**

In 2018 the Psychiatry Ward Review Safety Checklist was created for ward reviews on the Trust electronic clinical recording system with the aim to improve the documentation of legal and safety information. In 2019 an audit was conducted to ensure compliance with the safety checklist and in 2022 a re-audit was conducted to evaluate the effectiveness of the ward review checklist. Both audits examined 6 questions: Have you discussed the legal status of the patient? Is the patient for resuscitation? Does the patient currently have capacity for admission? Does the patient demonstrate deteriorating health? Does the patient have any physical health concerns? Review indication, current dosage and side effects of medications.

**Methods:**

A retrospective case note review of three ward round assessments of a sample of 25 patients. First male and first female admission of the month to Tower Ward (Landermere Centre, Clacton On Sea) were selected over the period from 1st December 2020 to 1st December 2021. Inclusion criteria: all patients. Exclusion criteria: None.

We maintained the same standards as the previous audit in 2018 and 2019: 80% completion.

**Results:**

12 male and 13 female patients were identified.

Q1. This was documented in 88% patients during the 1st week, and in 100% patients in mid-point stay and pre-discharge. In 2019 it was documented in 93% of the cohort.

Q2. This was documented in 42% in the 1st week, in 53% patients in midpoint and 45% in pre-discharge review. In 2019 this was recorded as 39% compliancy.

Q3. This was documented in 92% in the 1st week and midpoint, and in 67% during the pre-discharge review. In 2019 capacity was only documented in 14% of the cohort.

Q4. It was directly mentioned in 100% patients in all three reviews. In 2019 this was recorded in 64% of cases.

Q5. It was documented in 92% in the 1st week and mid-point review, and in 88% of the cohort in the pre-discharge review. In 2019 it was recorded for 69% of the cohort.

Q6. The information was included in 88% of the cohort during the 1st week, in 83% in mid-point and 75% in the pre-discharge review. In 2019 it was recorded for 81% of the cohort.

**Conclusion:**

Compared to the 2019 audit the overall compliance with the documentation was satisfactory (over 80%) in all audited points with the exception of question 2 regarding resuscitation status for all audited weeks (40–50%).

